# When Stroke Mimics a Subthalamic Lesion: An Unusual Presentation of Hemichorea-Hemiballismus

**DOI:** 10.7759/cureus.95663

**Published:** 2025-10-29

**Authors:** Akshayaa K Aggarawal, Mansoor Gazi

**Affiliations:** 1 Internal Medicine, Walsall Manor Hospital, Walsall, IMN; 2 Acute/Internal Medicine, Walsall Healthcare NHS Trust, Walsall, GBR

**Keywords:** brain vascular disease, chorea-ballism, stroke, thalamus stroke, vertebral artery stenosis

## Abstract

Hemichorea-hemiballismus (HCHB) is a rare hyperkinetic movement disorder characterized by involuntary, irregular, and forceful flinging movements affecting one side of the body. It most often occurs as an uncommon manifestation of ischemic stroke. Traditionally linked to contralateral subthalamic nucleus lesions, recent reports have shown that infarcts in other regions, such as the striatum, thalamus, cortex, and subcortical white matter, can also produce this presentation. We report a case of acute-onset right-sided hemichorea in an elderly female, secondary to a lacunar infarct in the left centrum semiovale. The patient was initiated on antiplatelet therapy for secondary stroke prevention and tetrabenazine for the symptomatic management of involuntary movements. This case underscores the diverse anatomical substrates and the evolving understanding of the pathophysiological mechanisms underlying HCHB.

## Introduction

Hemichorea-hemiballismus (HCHB) is an uncommon hyperkinetic movement disorder, clinically characterized by abrupt, irregular, and involuntary flinging or choreiform movements, typically confined to one side of the body [1]. These movements often involve both upper and lower extremities ipsilaterally and are distinguished by their coarse, wide-amplitude nature [2]. Epidemiologically, post-stroke movement disorders are rare, occurring in fewer than 1% of patients, with HCHB representing the most frequent subtype, particularly after ischemic events [3,4].

Traditionally, HCHB has been attributed to lesions of the contralateral subthalamic nucleus (STN), a pivotal structure within the basal ganglia-thalamocortical inhibitory circuitry. The STN exerts excitatory glutamatergic input to the globus pallidus interna (GPi), which in turn mediates GABAergic inhibition of thalamocortical motor output. Ischemic disruption of the STN reduces GPi activation, thereby attenuating thalamic inhibition and resulting in hyperkinetic motor manifestations [1].

However, accumulating evidence demonstrates that HCHB is not confined to subthalamic pathology. Vascular lesions involving the striatum, thalamus, cerebral cortex, subcortical white matter, and cerebellum have been implicated, underscoring the distributed and complex organization of motor inhibitory networks [5-7]. Rarely, an isolated centrum semiovale infarction has been reported as a cause of hemichorea [5]. Similarly, cortical and thalamic infarcts are increasingly recognized as atypical but clinically relevant substrates [6].

Beyond its role in motor regulation, the STN also contributes to cognitive and behavioral functions, highlighting its broader neurobiological importance [1]. While HCHB may be self-limiting, it can be severely disabling in the acute phase. Timely recognition, neuroimaging, and initiation of symptomatic pharmacologic therapy, typically dopamine-depleting or dopamine-receptor-blocking agents, are essential, alongside standard secondary stroke prevention strategies [3].

We report the case of an 80-year-old female who developed acute right-sided HCHB secondary to lacunar infarcts in the left centrum semiovale, in the setting of cerebral small vessel disease in the brain. This case expands current understanding of the diverse vascular and anatomical substrates underlying HCHB and underscores the importance of considering atypical infarct locations during clinical evaluation.

## Case presentation

An 80-year-old female with a history of dilated cardiomyopathy (ejection fraction 45%), hypertension, and a known hepatic cyst presented with a one-day history of abnormal involuntary movements involving the right side of her body. The movements had a sudden onset, were choreiform in nature, and were severe enough to interfere with mobility and daily activities. They predominantly affected the right upper and lower limbs and extended to the right side of the face. Throughout the episodes, the patient remained fully conscious, alert, and oriented, with intact comprehension and ability to follow commands. Vital signs were stable: blood pressure 130/80 mmHg, heart rate 80 beats per minute, respiratory rate 16/min, and normal oxygen saturation on room air, with no fever. There was no history of seizures, incontinence, post-ictal confusion, fever, cough, abdominal pain, or vomiting.

On examination, the patient was cooperative, fully oriented, and able to execute commands appropriately. Neurological evaluation revealed preserved muscle strength in all four extremities, with no sensory deficits, cranial nerve palsy, rigidity, or cerebellar signs. The involuntary movements were hyperkinetic, irregular, and ballistic in character, consistent with HCHB.

**Figure 1:**
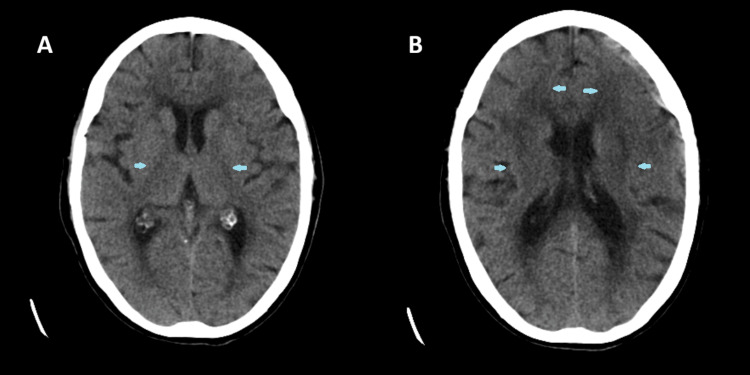
CT head for the patient Non-contrast axial CT images of the brain show no evidence of acute intracranial hemorrhage or large territorial infarction. The scans (A) and (B) demonstrate mild diffuse cerebral atrophy and chronic small-vessel ischemic changes (marked in blue arrows).

A CT head performed at the time of admission showed no evidence of acute infarction or hemorrhage, with periventricular hypoattenuation consistent with chronic small vessel disease (Figure 1).

**Figure 2:**
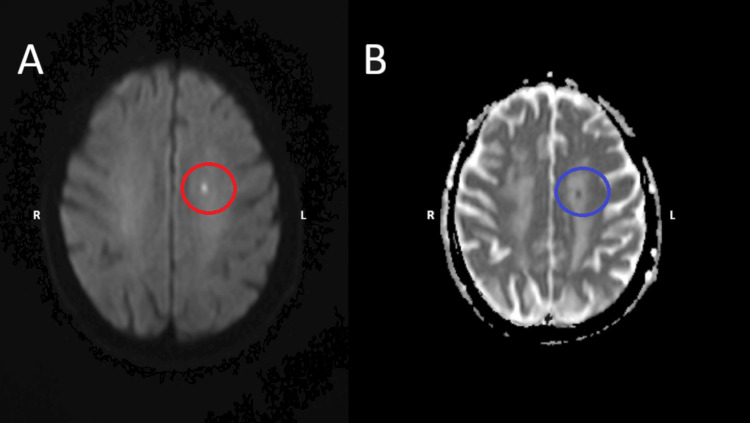
MRI images confirming the lesion Axial MRI brain images demonstrating a focal area of diffusion restriction in the left centrum semiovale, consistent with an acute lacunar infarct.  (A) The DWI image (lesion encircled in red). (B) The ADC image (lesion encircled in blue). DWI: diffusion-weighted imaging, ADC: apparent diffusion coefficient The images have been marked with right (R) and left (L) for orientation.

Subsequent MRI of the brain revealed tiny acute lacunar infarcts in the left centrum semiovale as the cause of the disease (Figure 2).

**Figure 3:**
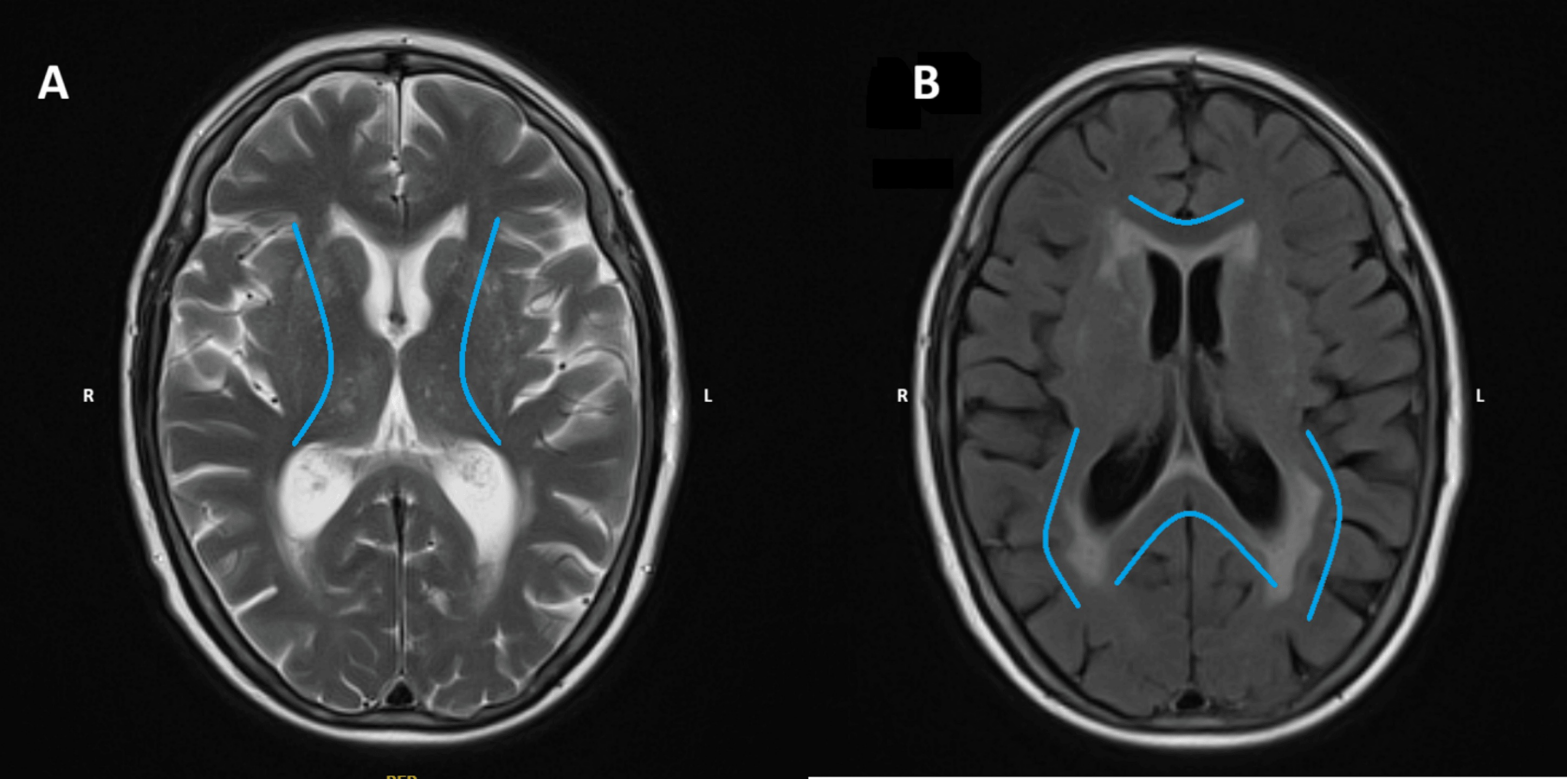
MRI head showing the chronic white matter changes Axial MRI brain images demonstrating chronic changes. (A) T2-weighted image. (B) Corresponding FLAIR sequence showing chronic white matter changes (in blue) FLAIR: fluid-attenuated inversion recovery The images have been marked with right (R) and left (L) for orientation.

Background white matter hyperintensities were noted, consistent with cerebral small vessel disease (Figure 3).

**Figure 4:**
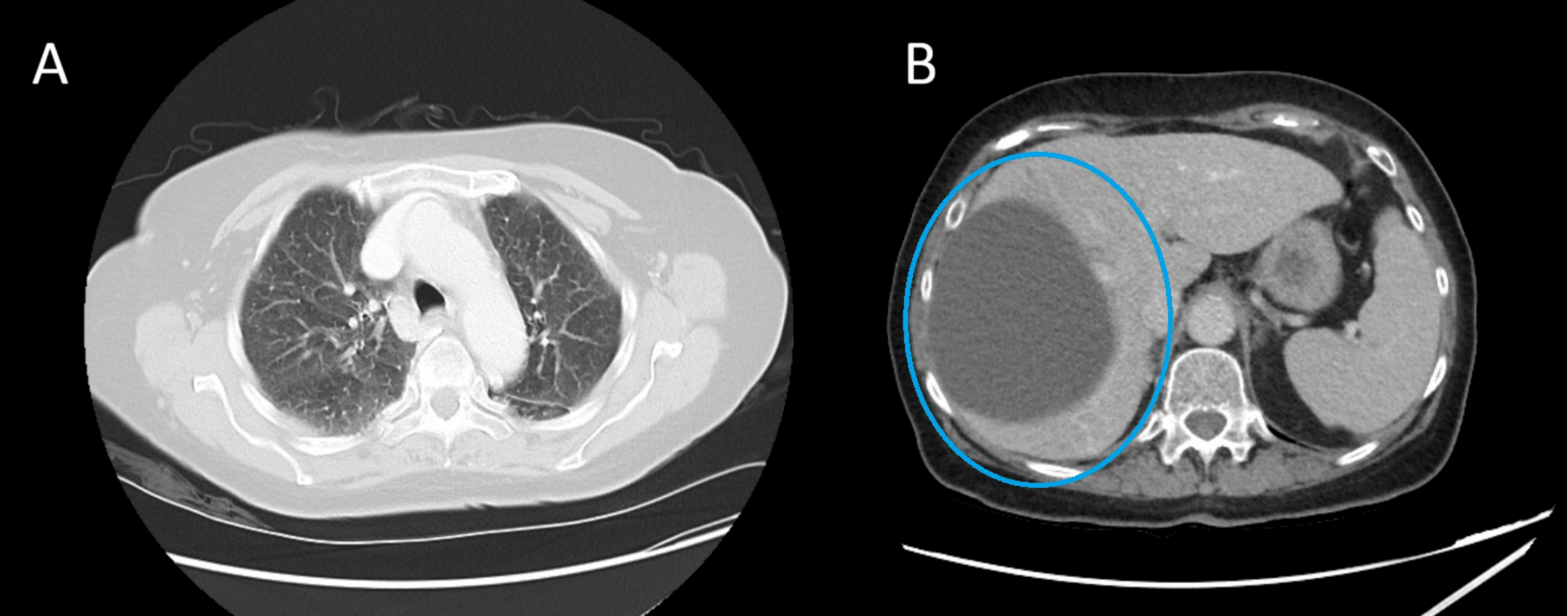
Representative contrast-enhanced chest and abdominal CT scan Contrast-enhanced axial CT images of the thorax and abdomen. (A) The thoracic section demonstrates normal lung fields with mild interstitial changes and no evidence of pulmonary embolism or focal consolidation. (B) The abdominal section reveals a large, well-defined, hypodense cystic lesion in the left hepatic lobe (circled in blue).

The CT of the thorax, abdomen, and pelvis was done, which showed a simple liver cyst and no neoplastic lesion (Figure 4). The cyst is asymptomatic with no further workup done. 

Initial blood investigations revealed no significant abnormalities. The complete blood count, liver and renal function tests, and infection markers were all within normal limits. Screening for secondary causes of movement disorders, including Wilson’s disease, thyrotoxicosis, and non-ketotic hyperglycaemic hemichorea (NKHHS) was done and negative (Table 1). The serum autoimmune disorders workup was negative (Table 2). Autoimmune encephalitis screening also yielded negative results (Table 3). Further evaluation for paraneoplastic syndromes showed no evidence of underlying malignancy (Table 4). 

**Table 1:** Serological screening tests TSH: thyroid-stimulating hormone, ANCA: anti-neutrophil cytoplasmic antibodies, NKHHS: non-ketotic hyperglycaemic hemichorea

Test	Result	Reference range	Interpretation
Serum copper	24.1	11.0-25.0 (umol/L)	Negative for Wilson disease
Serum caeruloplasmin	0.41	0.20-0.60 (g/L)	
TSH	1.05	0.35-4.94 (miu/l)	Negative for thyrotoxicosis
Thyroid peroxidase antibody	<3	4-1542 IU/ml	
Myeloperoxidase (MPO) ANCA	<0.2	0-3.5 (IU/ml)	Negative for autoimmune disorder
Proteinase 3(PR3) ANCA	<0.6	0-2 (IU/ml)	
Ammonia	31	<40 (umol/L)	Negative for hepatic encephalopathy
Random plasma glucose	5.5	3.9-6.0 (mmol/L)	Negative for NKHHS

**Table 2:** Serological tests for autoimmune diseases

Test	Result
Anti-nuclear antibody screen	Negative
Gastric parietal cell antibody	Negative.
Mitochondrial antibody	Negative
Smooth muscle antibody	Negative
Liver kidney microsomal antibody	Negative

**Table 3:** Autoimmune encephalitis screen AMPA: α-amino-3-hydroxy-5-methyl-4-isoxazolepropionic acid receptor, LGI-1 antibody: leucine-rich glioma-inactivated 1 antibodies, GABAB 1/2: gamma-aminobutyric acid type B receptor subunit 1/2, CASPR2: contactin-associated protein-like 2

Test	Result
AMPA1 receptor antibody	Negative
AMPA2 receptor antibody	Negative
LGI-1 antibody	Negative
N-methy D-asparate (NMDA) receptor antibody	Negative
GABAB1/2 receptor antibody	Negative
CASPR2 antibodies	Negative

**Table 4:** Serological paraneoplastic panel CV2/CRMP5: collapsin response mediator protein 5, MA2: para-neoplastic Ma family member 2, SOX1: SRY-box transcription factor 1, ZIC4: zinc finger protein of the cerebellum 4, GAD 65: glutamic acid decarboxylase 65, Tr (DNER): Tr antigen-delta/notch-like epidermal growth factor-related receptor

Test	Result
Anti-CV2/CRMP5	Negative
Anti-Ma2	Negative
Anti-amphiphysin	Negative
Anti-Ri	Negative
Anti-Yo	Negative
Anti-Hu	Negative
Anti-recoverin	Negative
Anti-SOX1	Negative
Anti-Titin	Negative
Anti-ZIC4	Negative
Anti-GAD 65	Negative
Anti-Tr (DNER)	Negative

Based on the clinical examination and diagnostic work-up, the patient was diagnosed with acute-onset right-sided hemichorea. Symptomatic management with tetrabenazine 25 mg was initiated, leading to partial improvement in the abnormal movements. Antiplatelet therapy with aspirin (300 mg for 14 days, followed by clopidogrel 75 mg for life) and a statin (atorvastatin 80 mg) was started in view of the acute lacunar infarcts to reduce the risk of future vascular events. The patient was reviewed by the physiotherapy team for supportive management and rehabilitation.

Upon stabilization, she was discharged on aspirin 300 mg, atorvastatin 80 mg, and tetrabenazine 25 mg, in addition to her pre-existing regimen of anti-hypertensive medication (lercanidipine 20 mg tablets and ramipril 1.25 mg) and for heart failure (dapagliflozin 10 mg tablets). She was advised to follow up in the transient ischemic attack (TIA) clinic for ongoing evaluation, vascular risk factor management, and neurologic monitoring.

## Discussion

HCHB is an uncommon hyperkinetic movement disorder most frequently observed in association with cerebrovascular disease. In the Lausanne Stroke Registry, which analyzed data from 2,500 patients, 29 individuals (1%) were identified with acute or delayed-onset movement disorders. Among these, the most frequent presentations were HCHB and hemidystonia, typically associated with strokes involving the basal ganglia and adjacent white matter within the middle cerebral artery territory [8]. Similarly, Dewey et al. examined 21 patients and found that stroke was the most common cause of movement disorders, accounting for nearly half of the cases (10 of 21), thereby reinforcing the vascular basis of HCHB [9].

Hemiballismus results from disruption of the basal ganglia inhibitory pathways, most commonly involving the subthalamic nucleus or globus pallidus internus (GPi). Damage to these structures reduces inhibitory output from the GPi and leads to disinhibition of the thalamus, specifically the ventral anterior and ventral lateral nuclei, causing excessive activation of the corticospinal and corticobulbar tracts and producing the characteristic, high-amplitude involuntary movements on the contralateral side. The basal ganglia regulate motor activity through two interconnected circuits: the direct pathway, which facilitates movement via D1 receptor-mediated inhibition of the GPi, and the indirect pathway, which suppresses movement through D2 receptor-mediated activation of the GPi via the subthalamic nucleus [1].

Classically, HCHB has been attributed to lesions of the contralateral subthalamic nucleus, where disruption of the basal ganglia-thalamocortical circuitry results in thalamic disinhibition and excessive motor output [1]. However, accumulating evidence indicates a broader anatomical spectrum, with infarcts involving the striatum, thalamus, cerebral cortex, subcortical white matter, and even the cerebellum reported as causative sites [5,6]. The present case adds to this growing body of literature by documenting hemiballismus secondary to a lacunar infarct in the centrum semiovale, a lesion location only rarely described in previous reports [5].

A wide spectrum of systemic conditions can manifest with movement disorders, including endocrine abnormalities, hepatic or renal dysfunction, hyperglycemia, and electrolyte disturbances. Although the exact mechanisms remain incompletely understood, these disorders are thought to arise from disruption of the basal ganglia-frontal motor cortex circuitry that finely regulates voluntary movement. When evaluating a patient with acute chorea or hemiballismus, it is important to consider both structural and metabolic causes. Among these, cerebrovascular disease and NKHHS are most frequently implicated [10-12]. While cerebrovascular lesions account for the majority of acute-onset cases, abnormalities in glucose metabolism and electrolyte balance are equally significant contributors to acute or subacute presentations. In particular, NKHHS tends to evolve over several days and may lead to either transient or persistent hyperkinetic movements [13].

Management of post-stroke hemiballismus is primarily symptomatic. Dopamine-depleting agents such as tetrabenazine, as well as dopamine receptor-blocking agents, have shown benefit in reducing involuntary movements [6]. Nonetheless, spontaneous improvement is not uncommon, with up to 50% of patients experiencing complete resolution within six months [9]. In the present case, tetrabenazine produced partial symptomatic relief, while antiplatelet and statin therapy targeted the underlying vascular pathology to reduce recurrence risk. Early physiotherapy and integration into a multidisciplinary stroke rehabilitation program further supported functional recovery.

This case highlights several key clinical insights. First, hemiballismus may occur from infarcts outside the classical subthalamic nucleus, including the centrum semiovale and cerebellum, emphasizing the broader anatomical spectrum of this disorder. Second, both small- and large-vessel disease may act synergistically to precipitate movement disorders following stroke. Finally, comprehensive multimodal imaging, particularly MRI with DWI (diffusion-weighted imaging) and ADC (apparent diffusion coefficient) images, is invaluable for identifying both acute and chronic vascular substrates responsible for atypical post-stroke presentations.

## Conclusions

This case highlights a rare presentation of HCHB secondary to lacunar infarcts in the centrum semiovale, underscoring that this disorder is not limited to subthalamic lesions. Infarcts in atypical regions, including the centrum semiovale, thalamus, and cerebellum, can disrupt motor control pathways and manifest with similar hyperkinetic movements. Comprehensive multimodal neuroimaging, including MRI with DWI and ADC, is essential to identify small vessel changes and acute infarcts for an appropriate diagnosis. 

Early recognition of post-stroke HCHB and a multidisciplinary approach combining symptomatic management (such as tetrabenazine for movement suppression) with secondary stroke prevention (antiplatelet therapy, statin use, and rehabilitation) are crucial for optimizing outcomes. This case adds to the growing evidence that HCHB can arise from diverse vascular territories, expanding the clinical understanding of post-stroke movement disorders.

## References

[1] Cabrero JA, De Jesus O (2025). Hemiballismus. StatPearls [Internet].

[2] Hawley J, Weiner W (2011). Hemiballismus: current concepts and review. Parkinsonism Relat Disord.

[3] Butnaru A, Schreiner TG, Constantinescu M (2024). Post-stroke hemiballismus: a series of two cases. Rom J Neurol.

[4] Tater P, Pandey S (2021). Post-stroke movement disorders. Neurol India.

[5] Yi J, Zhang L, Zhang T, Li J, Zhang Y, Zhou M (2023). Cerebral infarction in centrum semiovale presenting with hemichorea: a case report and literature review. Front Neurol.

[6] King B, Jamil H, Dekhne A, Bajwa D, Nolte J, Ferguson P, Inam SHA (2024). Pure sensory thalamic stroke presenting as hemiballismus: a case report. Cureus.

[7] Wessel JR, Diesburg DA, Chalkley NH, Greenlee JDW (2022). A causal role for the human subthalamic nucleus in non-selective cortico-motor inhibition. Curr Biol.

[8] Ghika-Schmid F, Ghika J, Regli F, Bogousslavsky J (1997). Hyperkinetic movement disorders during and after acute stroke: the Lausanne Stroke Registry. J Neurol Sci.

[9] Dewey R (1989). Hemiballism-hemichorea. Arch Neurol.

[10] Ticona J, Zaccone V, Zaman U (2020). Hemichorea-hemiballismus as an unusual presentation of hyperosmolar hyperglycemic syndrome. Am J Med Case Rep.

[11] Marques JS, Monteiro N, Nunes A (2018). Hyperglycaemic hemichorea. Eur J Case Rep Intern Med.

[12] Ryan C, Ahlskog JE, Savica R (2018). Hyperglycemic chorea/ballism ascertained over 15 years at a referral medical center. Parkinsonism Relat Disord.

[13] Yu JH, Weng YM (2009). Acute chorea as a presentation of Graves disease: case report and review. Am J Emerg Med.

